# Mechano-electric response of tribopositive, fluoropolymer-free polyvinyl acetate nanogenerators in multiple contact electrification

**DOI:** 10.1016/j.isci.2026.117808

**Published:** 2026-10-12

**Authors:** Petr Slobodian, Pavel Riha, Berenika Hausnerova

**Affiliations:** 1 Department of Physics and Materials Engineering, Tomas Bata University in Zlin, Zlin, Czech Republic; 2 Department of Production Engineering, Faculty of Technology, Tomas Bata University in Zlin, Zlin, Czech Republic; 3 Centre of Polymer Systems, University Institute, Tomas Bata University, Zlin, Czech Republic

**Keywords:** polymer adhesives, contact electrification, triboelectric nanogenerator, electrostatic induction, strain-gradient effects

## Abstract

Polyvinyl acetate (PVAc) is a highly polar, fluorine-free polymeric adhesive that combines strong interfacial adhesion with good processability. Despite this, PVAc has rarely been investigated as an active layer in mechano-electric nanogenerators, even though its strong adhesion facilitates effective interfacial contact, thereby eliminating the need for auxiliary conductive layers. Electrical measurements revealed that the electrical output of PVAc arises from the interplay of triboelectric charge transfer, electrostatic induction driven by residual polarization, and deformation-induced interfacial polarization. The nanogenerators achieved an open-circuit voltage up to 350 V, a short-circuit current density of 1.8 μAcm^−2^, and power density of 0.07 mWcm^−2^ at a maximum power point of 4.4 MΩ. Stable output over 10^4^ cycles demonstrated that PVAc is a suitable polymer for a new generation of fluoropolymer-free nanogenerators and flexible pressure sensors.

## Highlights

• Tribopositive PVAc enables fluoropolymer-free mechano-electric harvesting
• Triboelectric, electrostatic, and deformation-related effects act cooperatively
• PVAc reaches open-circuit voltage up to 350 V and remains stable over 10,000 cycles
• Pre-pressure and film thickness govern static-contact performance

## Graphical abstract

**Figure:**
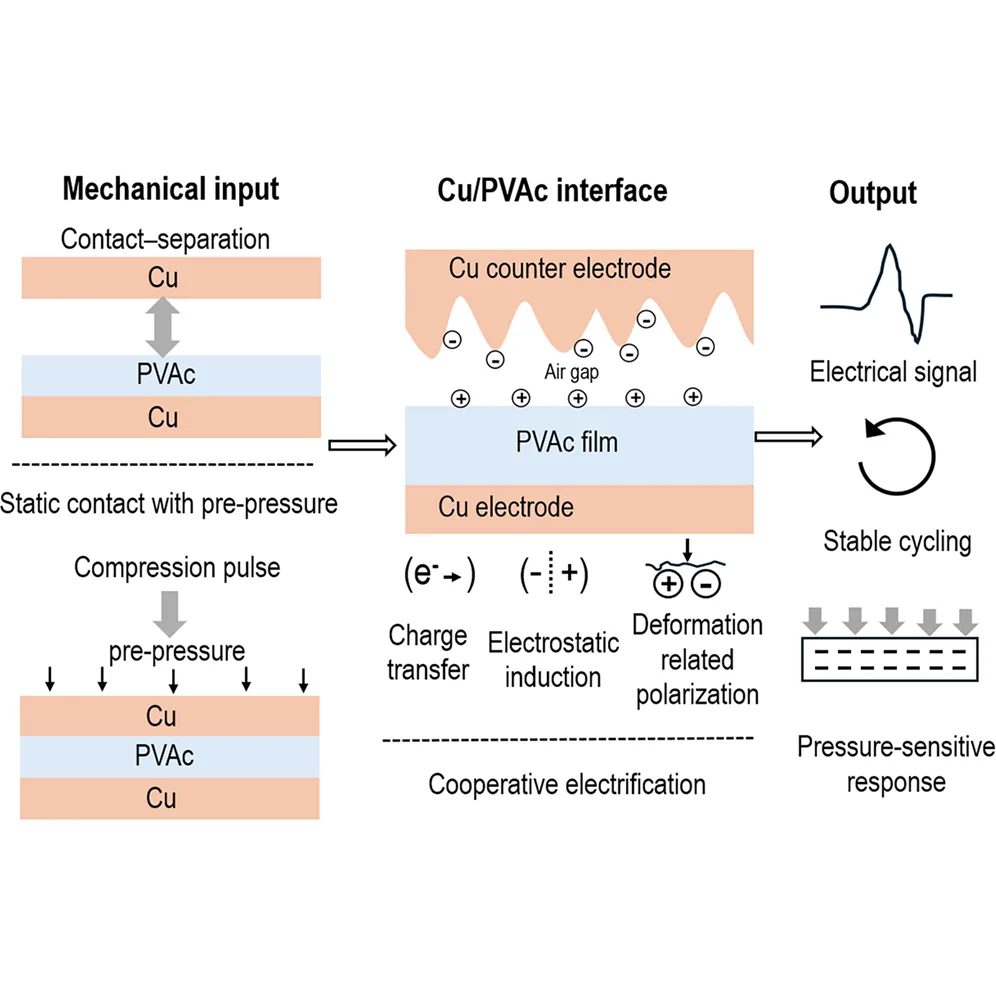

## Teaser

Applied sciences; Materials science

## Introduction

Polyvinyl acetate (PVAc) is commonly used in construction materials as an adhesive component of cement and plaster mixtures, paints, protective layers, lamination and adhesive films in the packaging industry, surface treatment of textiles, controlled drug release systems, etc. Despite promising relevant physical properties, PVAc has rarely been considered for mechano-electric conversion.

A large proportion of high-output triboelectric nanogenerators (TENGs) rely on strongly tribonegative fluoropolymers (e.g., polytetrafluoroethylene [PTFE]- or polyvinylidene fluoride [PVDF]-based layers) because they provide a stable negative charge and high surface potential. In this context, tribopositive polymers remain relatively less explored as primary active layers. PVAc can be easily molded, printed, or cast into sensor films. Unlike fluoropolymers, PVAc does not contain hazardous per- and polyfluoroalkyl substances, which makes it attractive for green energy harvesting. The adhesion parameters of PVAc (contact angle 60.6°, critical surface tension 35.3 ± 2.08 mN/m, and solid surface free energy 38.5 ± 2.75 mN/m)^1^ in comparison to those of PVDF, with the corresponding values^2^ of 93°, 31.6 ± 5.83 mN/m, and 31.5 ± 5.29 mN/m, result from stronger intermolecular forces (hydrogen bonding and dipole interactions) present in PVAc, while PVDF is more hydrophobic owing to its fluorine content.^3^

Novel wearable electronics prepared through roller printing technology based on the adhesion differences of semi-liquid metal on cotton fabrics and PVAc show that PVAc significantly enhances adhesion between semi-liquid metal and fabric substrates. Its selective adhesion to PVAc, but not to raw cotton fabric, enabled the precise patterning of conductive circuits (corresponding contact angles were reduced by almost 100°; the adhesion force on PVAc adhesive was approximately seven times higher than that on a bare fabric).^4^

Here, we investigate the contact electrification behavior of PVAc in contact with copper (Cu) using different experimental setups. Verners et al.^5^ included PVAc among six model polymers to verify the roughness-dependent contact electrification mechanism. The measured charge density for PVAc was only 0.10 nCcm^−2^, and no device-level characterization was performed. The present work is therefore, to the best of our knowledge, the first systematic study of PVAc as a functional triboelectric material for nanogenerator applications.

Besides triboelectricity, the material polarization when subjected to homogeneous deformation—piezoelectricity—has been studied extensively in the last decade.^6^^,^^7^^,^^8^^,^^9^^,^^10^^,^^11^ The weak piezoelectric properties in PVAc have been reported by Ounaies et al.^12^ On the other hand, flexoelectricity, a phenomenon far more general than piezoelectricity, as it applies to all dielectrics rather than just those with non-centrosymmetric space groups, is not being taken into account at all in the context of PVAc. The concept of flexoelectricity was originally introduced by de Gennes and Prost in the context of liquid crystals, where polarization arises from gradients of molecular orientation.^13^ Flexoelectric effects can be employed to control the internal electric field, manipulate transport properties, and develop actuators and energy harvesters.^14^ As a soft polymer, PVAc can exhibit strain gradients when deformed, leading to a strain-gradient-induced polarization. Mechanical deformation creates a charge asymmetry that can be used for low-frequency vibration-energy harvesting. Unlike piezoelectric materials, PVAc does not require permanent dipole alignment, which makes it easy for sensor applications.

Recently, it has been suggested that flexoelectric charges arising due to strain gradients lead to significant surface potential differences, which can act as drivers for triboelectric charge separation and transfer.^15^ It has been experimentally demonstrated that the coupling between the flexoelectric and triboelectric effects at the nanoscale in crystalline dielectric materials can affect measurements in contact-separation mode.^16^ Another study shows how flexoelectricity results in a fluctuating surface electric potential when elastic solids with random roughness are squeezed into triboelectric contact. When the flexoelectric potential induces surface charge distributions, it contributes to triboelectricity.^17^^,^^18^^,^^19^ Šutka et al.^20^ also admitted that triboelectricity and flexoelectricity may be interconnected. In complex mechanical systems involving soft interfaces, such as TENGs, multiple electromechanical contributions, including triboelectric, flexoelectric, and electrostatic induction phenomena, can coexist and interact, as recently reviewed by Olson and Marks.^21^

In this study, PVAc is used in an energy harvester, where triboelectric charge transfer, electrostatic induction, and deformation-related interfacial polarization can coexist. Contact-separation, air-gap measurements, and static-contact (pre-pressure) modes are used to investigate these contributions. Although PVAc has a relatively low dielectric permittivity compared to the typical high permittivity of materials used for mechano-electric conversion, its mechanical softness relative to the much higher elastic modulus of the Cu electrode, together with the surface micro-roughness of the electrode, may result in a combined electrification, which makes it ideal for wearable vibration sensors or flexible pressure-sensitive devices that require mechanical stability.

## Results

Dieletric analysis (DEA) (Figure 1) revealed a weak frequency dependence of the relative permittivity (ε′), with values slightly decreasing from 5.4 to 5.0 over the frequency range of 1 Hz–10 MHz. The dielectric loss tangent (tan δ) remained rather low (<0.01), indicating low energy dissipation and stable dielectric polarization.

**Figure 1:**
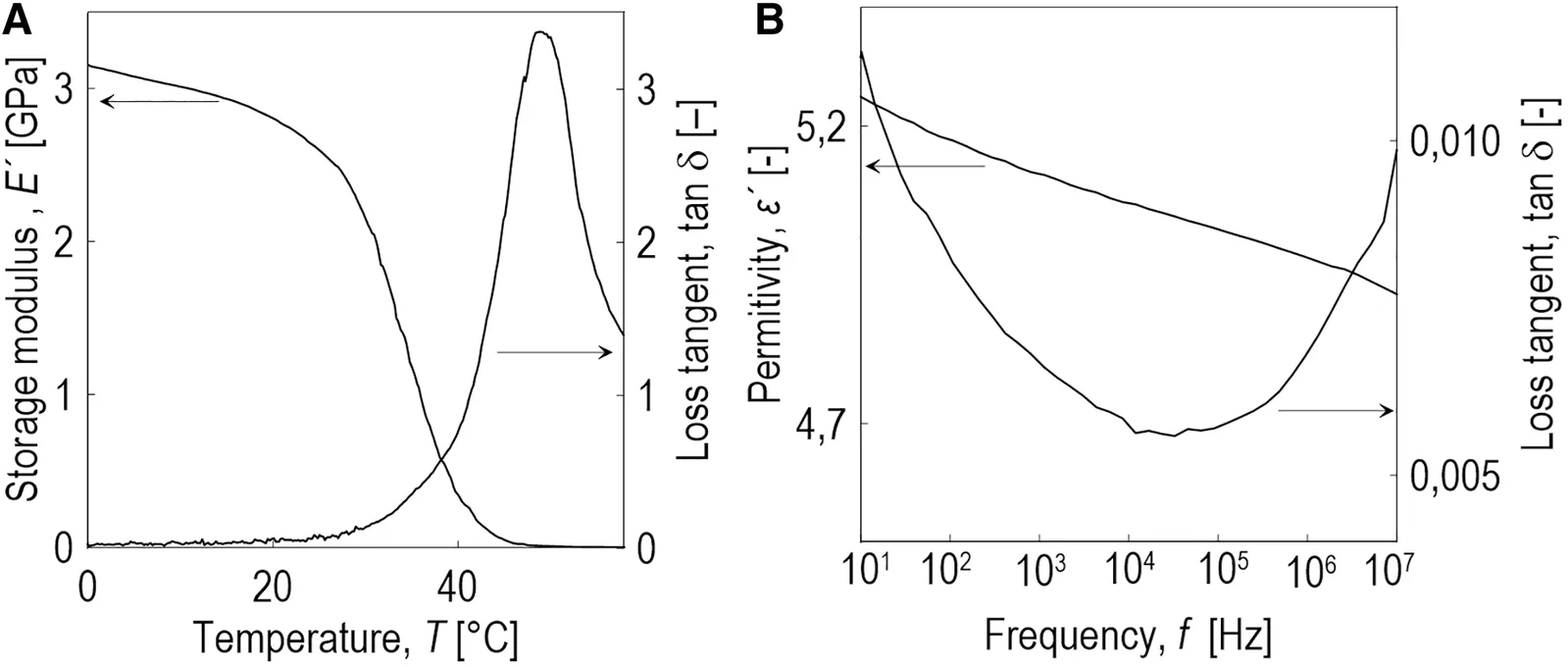
Thermomechanical and dielectric properties of the PVAc film Temperature dependence of (A) storage modulus and loss tangent of PVAc (DMA, tensile mode at 1 Hz) and (B) frequency dependence of dielectric permittivity and loss tangent (DEA, 1 Hz to 10 MHz).

Dynamic mechanical analysis (Figure 1) revealed a characteristic viscoelastic transition of the PVAc film; the storage modulus E′ decreased significantly from 2,890 MPa at 10°C, where the material was in the glassy state, to 10 MPa at 50°C, corresponding to the rubbery plateau. The glass transition region was clearly reflected in this decrease, with the evaluated glass transition temperature of 34.4°C identified as the inflection point on the storage modulus curve. The tan δ exhibited a maximum at 48.5°C.

The electrification modes are schematically shown in Figure 2. During the contact-separation mode, the PVAc film and Cu electrode surface formed a tribopair, which induced electron flow between the electrodes in the contact-separation process. According to triboelectric polarization, the PVAc polymer has the tendency to gain a positive charge (lose electrons), and the Cu foil tends to charge negatively (gain electrons). Deformation-driven interfacial polarization effects can occur in many dielectric/metal contacts when the dielectric is mechanically deformed in a non-uniform way. In our system, the Cu counter electrode was relatively rough with Sz = 3.827 μm (Sa = 0.482 μm and Sq = 0.582 μm), while the surface of the PVAc film was relatively flat with Sa = 0.029 μm, Sq = 0.038 μm, and Sz = 1.063 μm. Importantly, a pronounced mechanical stiffness difference exists between Cu (Young’s modulus ∼110 GPa) and PVAc (∼3 GPa in the glassy state). During compression, the rigid Cu asperities can locally indent a significantly softer PVAc layer. Although the strain distribution was not directly measured, such stiffness contrast makes the formation of localized deformation fields and strain gradients near the interface mechanically plausible. These strain gradients may influence the interfacial charge and potential distribution. Such strain-gradient-related polarization would be consistent with a flexoelectric-type contribution reported in the literature.^22^

**Figure 2:**
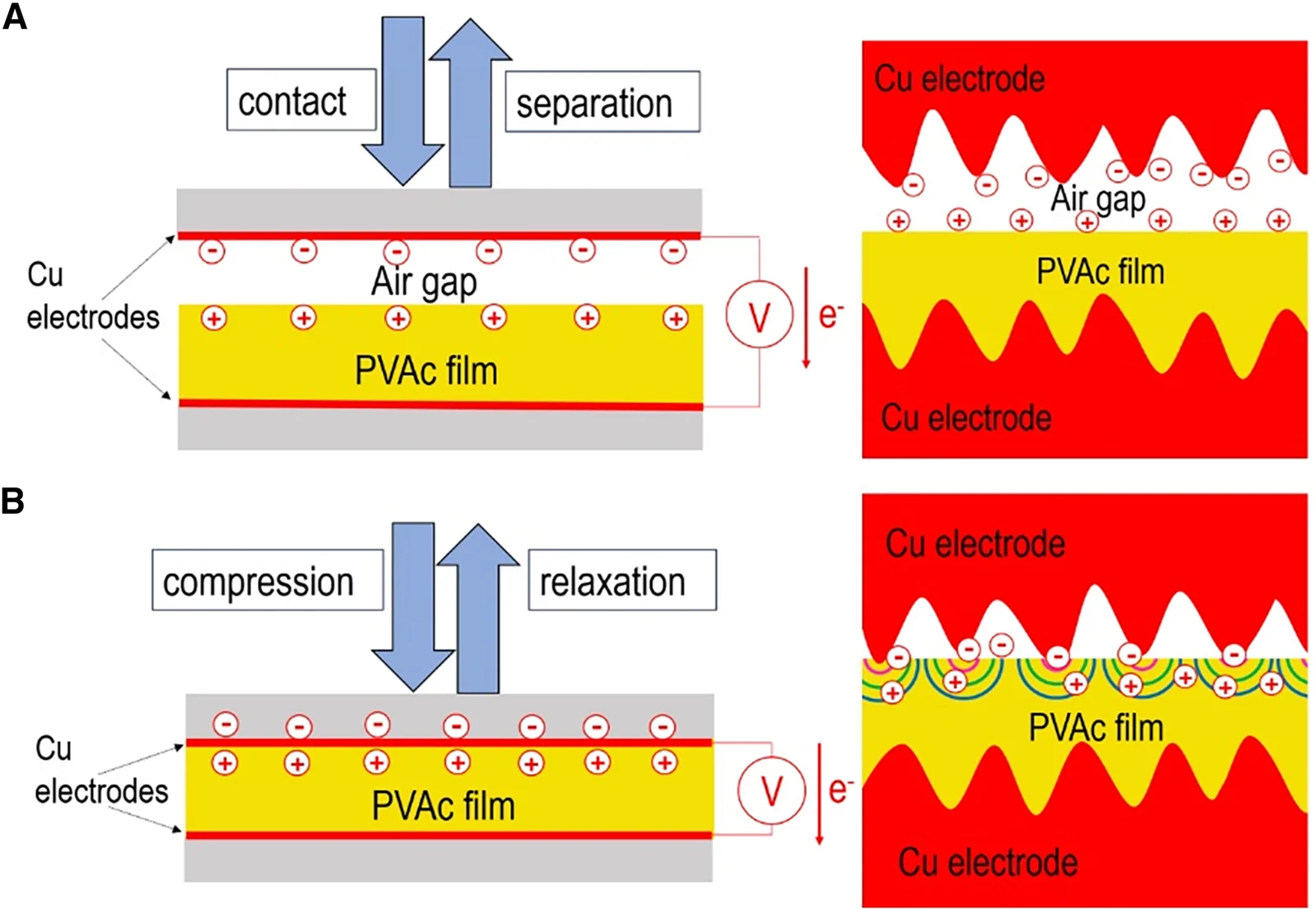
Schematic representation of the operating principles of the PVAc/Cu nanogenerator (A) Contact–separation mode. (B) Static-contact pre-pressure mode.

Measurements in contact-separation mode revealed an open-circuit voltage peak above 160 V lasting 5 ms, as shown in Figure 3. After the separation of the PVAc and Cu layers, the reverse exchange of electrons occurred resulting in an electric voltage of approximately −44 V. The recorded voltage transition reflects a rapid redistribution of charges within the device/electrode system through the external circuit during a single impact event. The initial peak sign is consistent with the known triboelectric tendency of PVAc to be positively charged relative to Cu under contact separation. After the first peak, a short opposite-polarity excursion can occur even when the interfaces are still in a macroscopic contact. We attribute this feature to the transient re-equilibration of the interfacial electric field and effective capacitance during a mechanically dynamic “contact window” (viscoelastic relaxation of PVAc, local asperity-level rearrangement, and partial rebound) in accordance with TENG literature, where the measured open-circuit voltage (or high-impedance voltage) can be decomposed into contributions from approach-induced electrostatic induction, charge separation at impact, and subsequent evolution of the separation gap and contact relaxation.^23^^,^^24^ This triboelectrification was reversible and stable, as shown for 10,000 cycles (Figure 3). The triboelectric charge density (TECD) was 16.3 ± 0.2 μCm^−2^.

**Figure 3:**
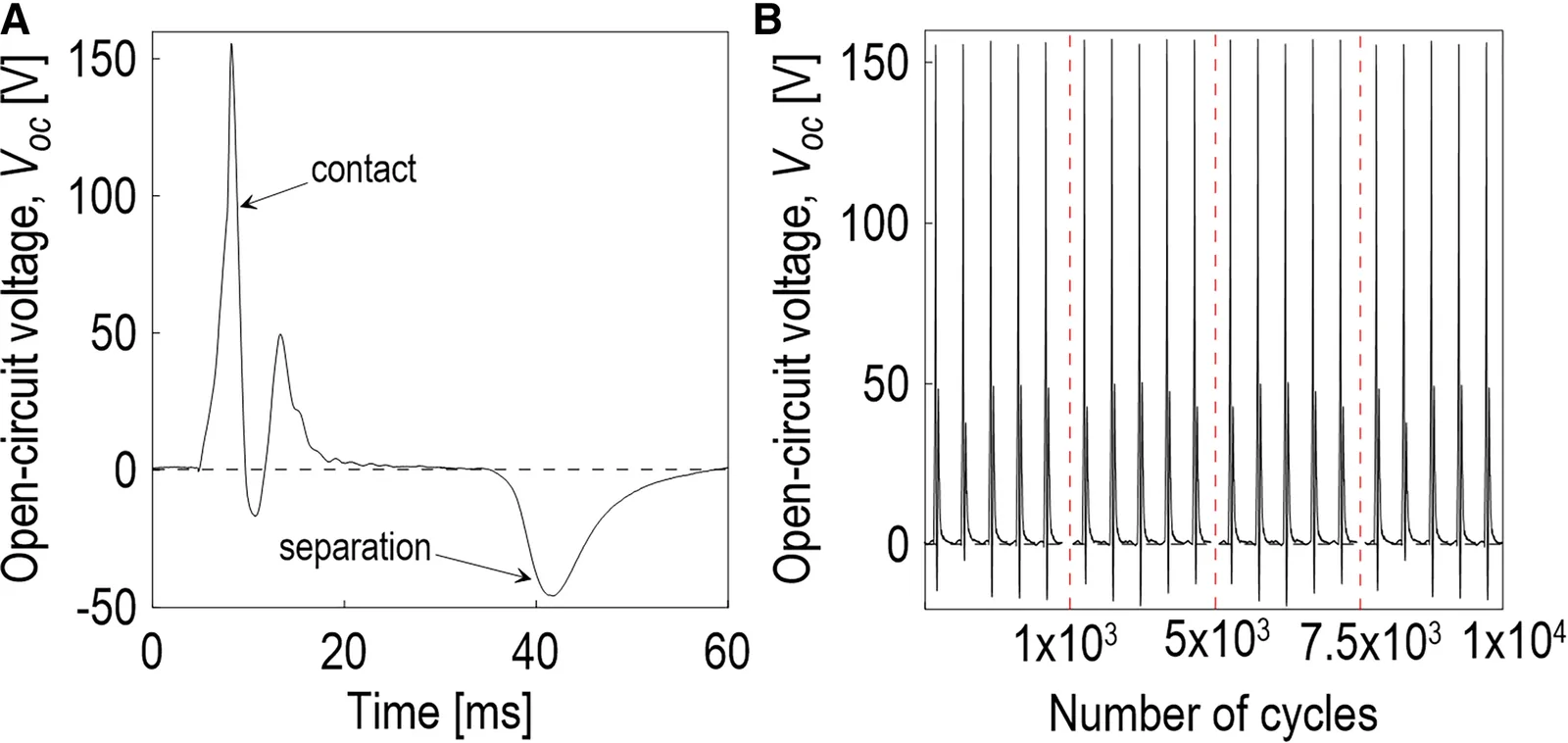
PVAc/Cu in contact-separation mode (A) Open-circuit voltage and (B) its stability up to 10^4^ cycles (1 Hz, 6.5 kPa, 18 cm^2^).

The resistive load-dependent electrical performance of the PVAc/Cu contact-separation nanogenerator measured at an applied pressure of 6.5 kPa and driving frequencies of 1 and 10 Hz is summarized in Figure 4. The device exhibits well-defined maximum power points at an external load resistance of approximately 4.4 (at 1 Hz) and 10 MΩ (at 10 Hz), indicating that the effective internal impedance of the PVAc-based generator is on the order of 10^6^–10^7^ Ω and that impedance matching is required to achieve maximum electrical power output and power density from the PVAc layer. At the optimum load of 4.4 MΩ, the PVAc/Cu device delivers a peak power density of about 0.071 mW · cm^−2^, consistent with the measured output voltage and current density values. Under lower resistance loading conditions (up to 10^5^ Ω), the generator provides substantially higher short-circuit currents of approximately 32 μA, corresponding to a current density of about 1.8 μAcm^−2^, demonstrating that the PVAc-based nanogenerator can supply appreciable current when operated in a low-impedance regime.

**Figure 4:**
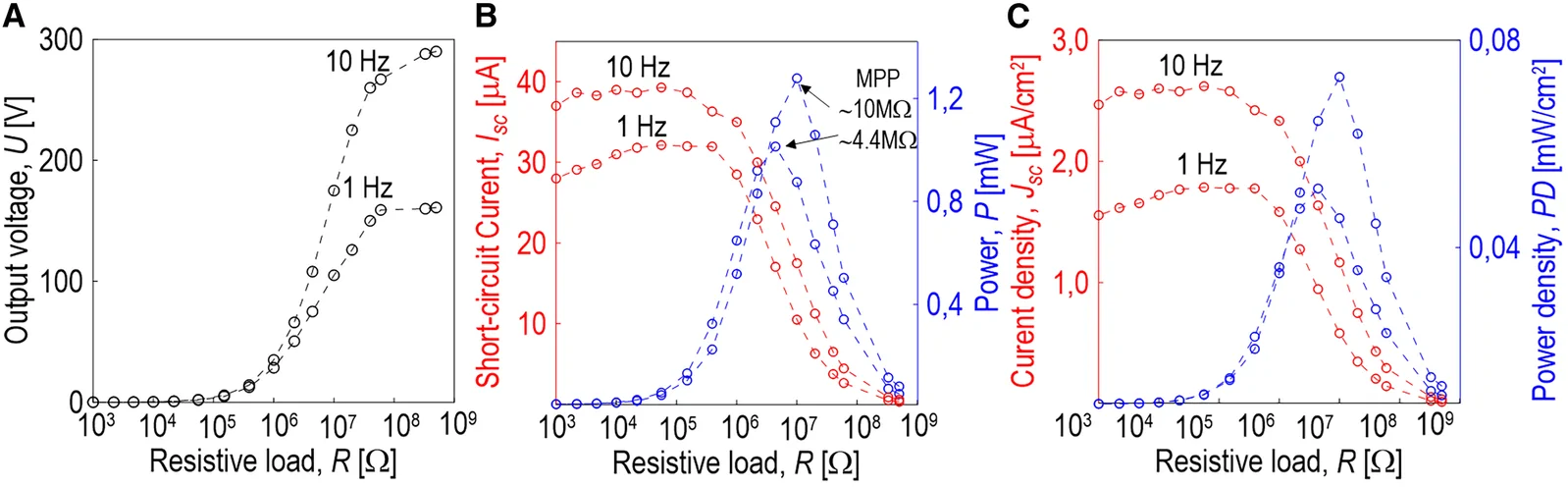
Resistive load and operating frequency determine electrical performance of PVAc/Cu nanogenerator in contact–separation mode Dependence of resistive load on (A) output voltage, (B) short-circuit current and power, and (C) current density and power density of PVAc/Cu TENG (vertical contact-separation, 1 and 10 Hz, 6.5 kPa, 18 cm^2^).

Electrification in static-contact (pre-pressure) mode as a contact response of PVAc and Cu electrodes to a pressure of 6.5 kPa and a contact pre-pressure of 0.4 kPa is shown in Figure 5. The open-circuit voltage peak was about 120 V and lasted 2 ms. This was followed by an oscillation of the electrical signal, apparently caused by an elastic vibration of the PVAc film lasting up to 15 ms. After releasing the 6.5 kPa pulse, an opposite-polarity voltage transient of approximately −28 V was observed, followed by a gradual return to the baseline. Since a constant pre-pressure of 0.4 kPa was maintained, macroscopic separation of the electrodes did not occur. The negative transient is attributed to the rapid elastic relaxation of the PVAc layer, which modifies the microscopic contact conditions at the rough Cu/PVAc interface and leads to a transient redistribution of interfacial charges. This electrification was also reversible, as shown over 10,000 voltage cycles (Figure 5). The effective transferred charge density was 5.9 ± 0.1 μCm^−2^, representing the net mechano-electrically generated charge under static-contact conditions.

**Figure 5:**
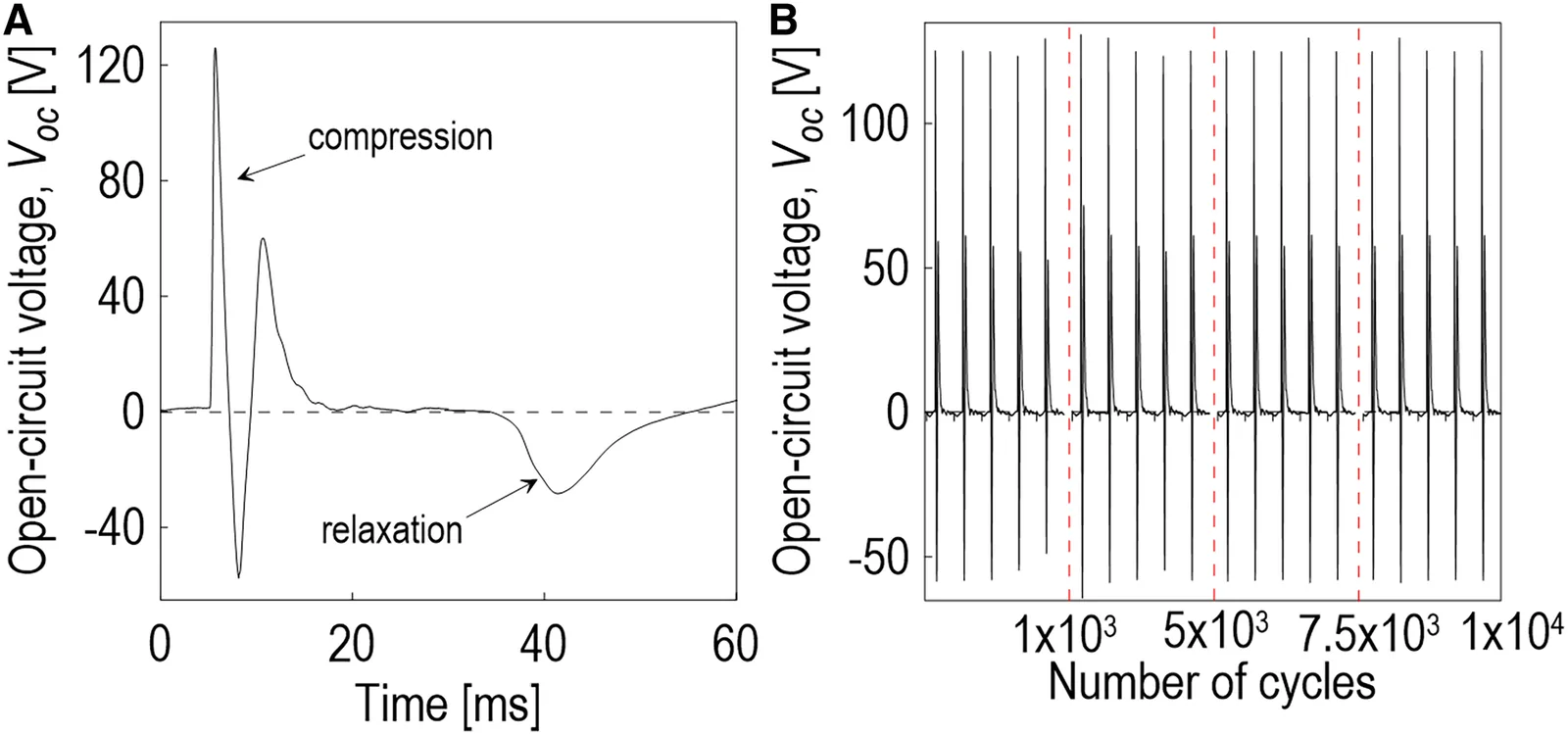
PVAc/Cu in static-contact (pre-pressure) mode generated by linear solenoid at contacting pre-pressure of 0.4 kPa (A) Open-circuit voltage and (B) its stability up to 10^4^ cycles (1 Hz, 6.5 kPa, 18 cm^2^).

The resistive load-dependent electrical performance of the PVAc/Cu nanogenerator operated in the static-contact pre-pressure mode is shown in Figure 6. Similar to the contact-separation regime, the device exhibits a well-defined maximum power point at an external load resistance of approximately 2.2 MΩ (in contrast to 4.4 MΩ for the contact-separation regime). At this optimum load, the pre-loaded PVAc/Cu configuration reaches a maximum power density of about 0.071 mWcm^−2^. Under low-resistance loading, short-circuit currents of approximately 47 μA were obtained, corresponding to a current density of about 2.6 μAcm^−2^ at negligible pre-pressure. As shown in Figure 6, increasing the static pre-pressure systematically reduces the output voltage, short-circuit current, current density, and power density. This trend is consistent with the suppression of microscopic deformation and interfacial charge redistribution at higher compressive loads. However, the position of the maximum power point remains nearly constant at approximately 2.2 MΩ across the investigated pre-pressure range.

**Figure 6:**
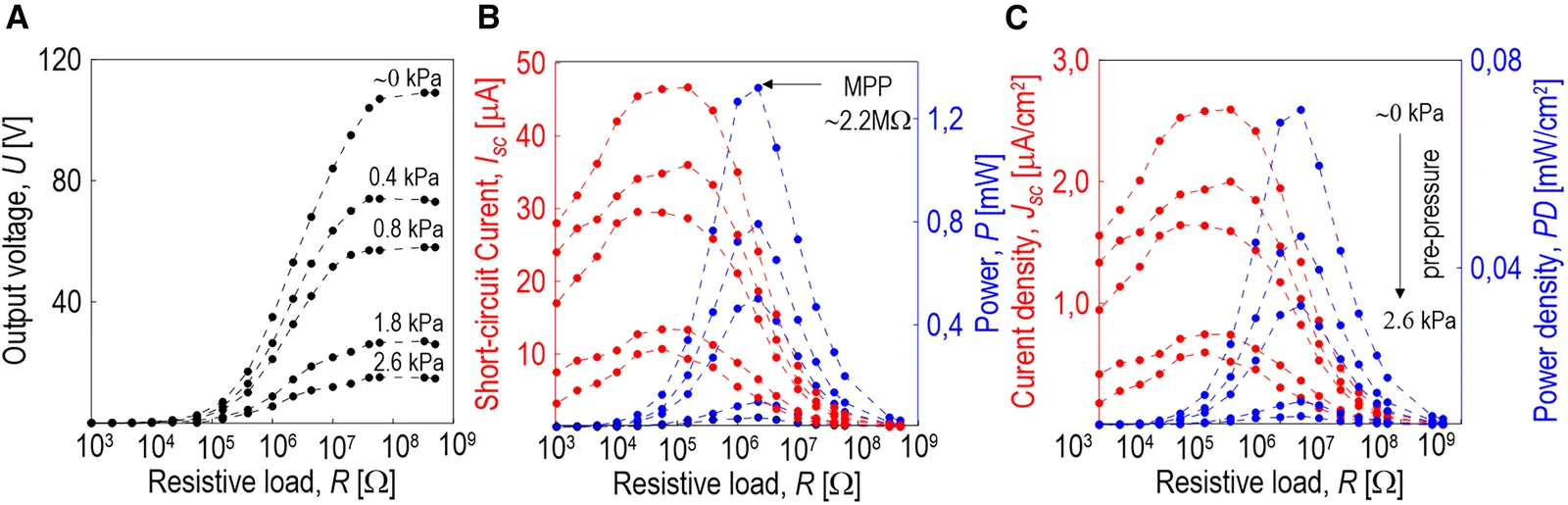
Static pre-pressure controls resistive-load-dependent electrical performance of PVAc/Cu nanogenerator Dependence of resistive load on (A) output voltage, (B) short-circuit current and power, and (C) current density and power density of PVAc/Cu TENG for various pre-pressures (static-contact mode, 1 Hz, 6.5 kPa, 18 cm^2^).

The effect of contact pre-pressures ranging from 0.1 to 7 kPa on the induced peak voltage before the application of a pressure pulse of 6.5 kPa (measured across a 1 MΩ load) is depicted in Figure 7. At a pressure of 0.1 kPa, the induced output voltage was 37 V; it then decreased to 26 V at 0.5 kPa, 11.7 V at 1.1 kPa, 5.3 V at 2.8 kPa, and to only 3.2 V at 7 kPa.

**Figure 7:**
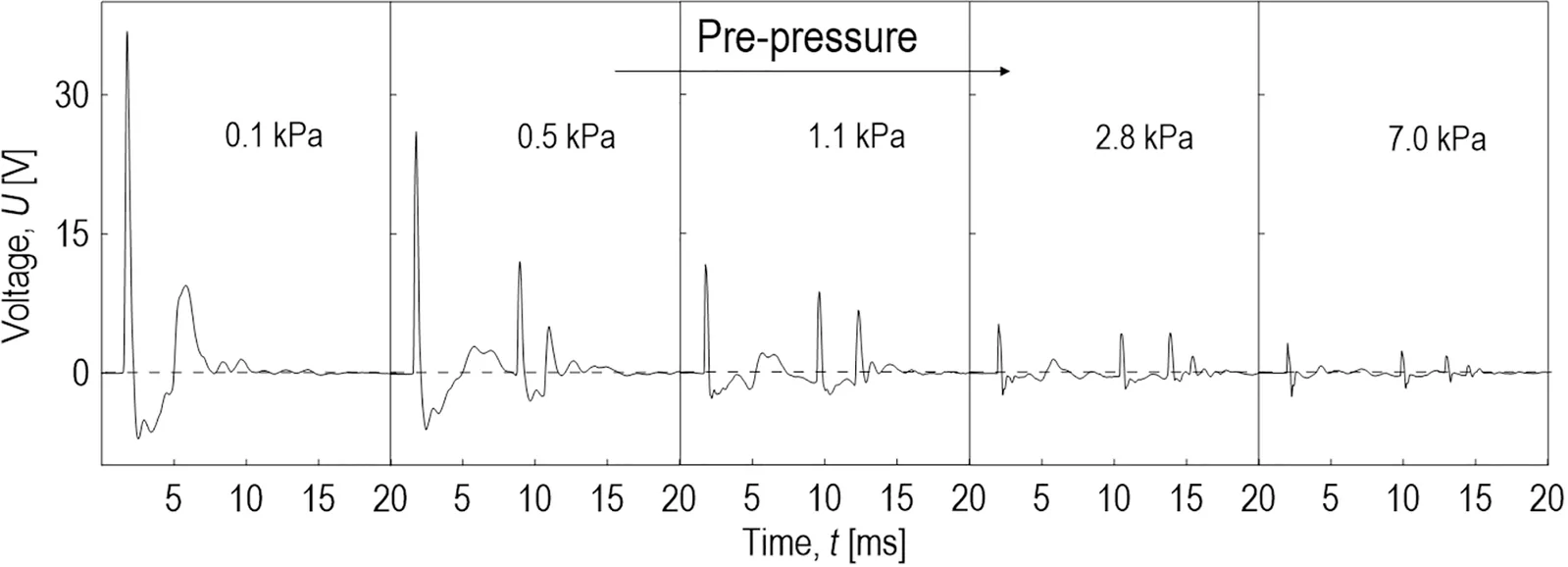
Pre-pressure-dependent output voltages of PVAc/Cu generated by linear solenoid (static-contact mode, 1 MΩ, 1 Hz, 6.5 kPa, 18 cm^2^)

A comparison of the two measurement modes (contact-separation and static-contact) depicted in Figure 8 reveals that while the position of the maximum output voltage is similar for both modes, the temporal evolution of the signals differs significantly. The curves are shown for three external loads (1, 2.2, and 4.4 MΩ). In the static-contact regime, measurements were performed at different pre-pressures, whereas in the contact-separation mode, no static pre-load was applied. In the contact-separation mode (red curves), the output voltage starts to increase during the approach phase due to electrostatic induction caused by the polarized PVAc layer, followed by a triboelectric contribution after mechanical contact and subsequent charge separation. The peak voltage increases with load resistance, reaching approximately 22.4 V at 1 MΩ, 42.3 V at 2.2 MΩ, and 65.6 V at 4.4 MΩ. At the point of contact, a notable shift occurs in the output-voltage curve, which can be attributed to rapid interfacial charge rearrangement and transient modification of the local polarization state of PVAc induced by mechanical interaction with the Cu electrode. The sharp voltage rise may also include contributions from deformation-related (flexoelectric-type) polarization associated with strain gradients near surface asperities. After reaching the maximum, the voltage reverses sign and gradually decreases, reflecting the evolving electrostatic configuration of the system.

**Figure 8:**
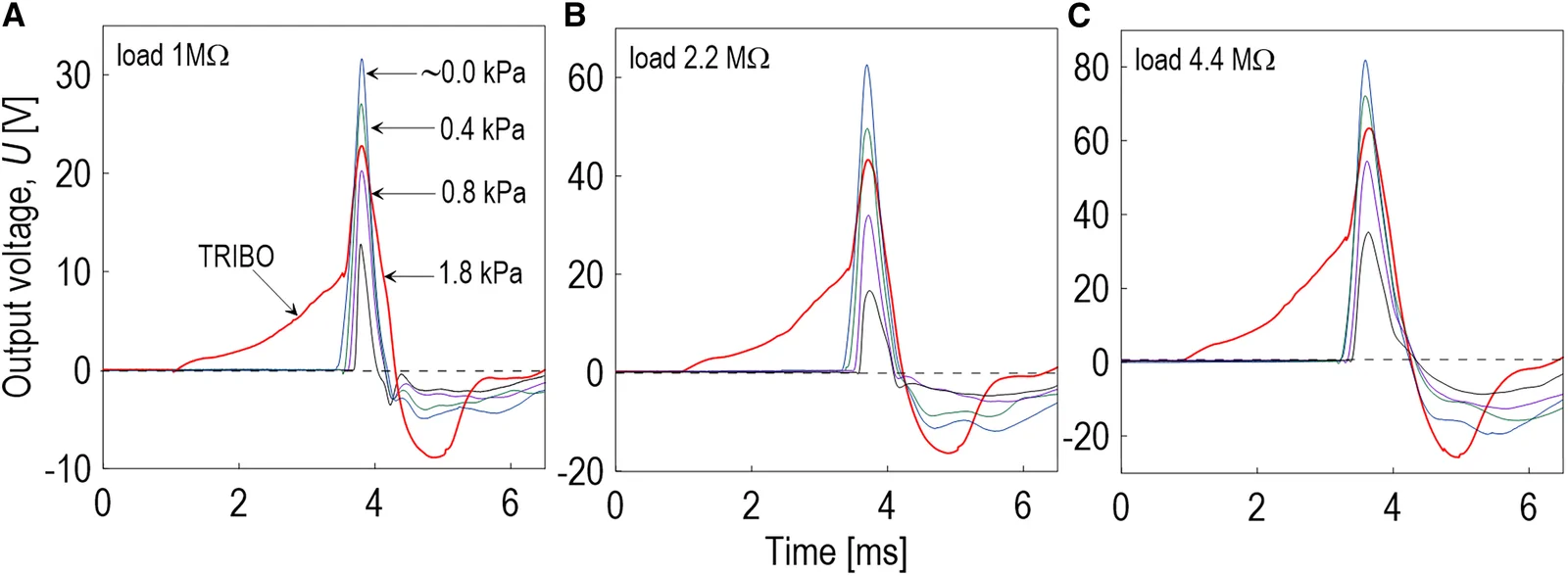
Comparison of output voltage curves obtained during contact-separation (red line) and static-contact (pre-pressure) measurement modes (1 Hz, 6.5 kPa, 18 cm^2^) for three external resistive loads (1 (A) and 2.2 (B) MΩ representing static-contact MPP, and 4.4 (C) MΩ representing contact-separation MPP)

In the static-contact configuration, the voltage peaks become markedly narrower compared to the contact-separation mode, reflecting the absence of the gradual electrostatic induction associated with the macroscopic approach. Since the electrodes are already in contact, the induction component related to changing separation distance is effectively suppressed, and the response is dominated by rapid interfacial charge redistribution during the pressure pulse. Interestingly, for low pre-pressures, the peak voltages exceed those obtained in the contact-separation regime. This behavior suggests that, at low static preload, enhanced localized deformation and interfacial charge modulation during the impact pulse may amplify the mechano-electric response beyond that achieved by macroscopic contact-separation alone. In contrast, for higher pre-pressures (0.8 and 1.8 kPa), the peak voltages decrease below the values observed in the contact-separation regime. At 1 MΩ, the maxima reduce to approximately 20 (0.8 kPa) and 12.4 V (1.8 kPa), at 2.2 MΩ to 31.6 and 16.3 V, and at 4.4 MΩ to 53.7 and 33.7 V, respectively. This progressive reduction with increasing static preload indicates that stronger initial compression suppresses microscopic contact modulation and limits the extent of interfacial charge redistribution during the pressure pulse.

Overall, the comparison demonstrates that static-contact operation does not merely suppress the triboelectric response but can either enhance or attenuate the output depending on the applied pre-pressure, highlighting the critical role of pressure-controlled interfacial mechanics in governing the mechano-electric response of the PVAc/Cu system.

In the static-contact (pre-pressure) mode (Figure 8), the materials did not move macroscopically relative to each other. As a result, the initial phase of the triboelectric peak owing to electrostatic induction was absent. However, when a pressure pulse was applied, a significant voltage was still generated. We hypothesize that this response originated from triboelectrification at the microscale owing to localized surface interactions. The interaction between the contacting materials is a key factor that influences charge generation. Given that Cu has a high modulus (110 GPa) and that PVAc is a significantly softer material (3 GPa), the Cu surface with its characteristic conical asperities could elastically deform the relatively smooth PVAc surface. This deformation, influenced by surface roughness, brought the contacting materials into closer proximity, which improved surface contact and led to lateral displacement or frictional interactions between the surfaces, promoting further triboelectrification. This increased proximity could also induce electrostatic polarization in the electrodes due to interfacial charge redistribution, even in the absence of macroscopic motion. On the other hand, this deformation might create strain gradients; thus, a possible flexoelectric polarization cannot be omitted.

The open-circuit voltage systematically increases with decreasing PVAc thickness (Figure 9). In the contact-separation mode, a maximum *V*oc of approximately 203 V was recorded for the thinnest tested film (0.3 mm). Increasing the thickness to 0.6 mm reduced *V*oc to ∼153 V, further to ∼116 V for 0.85 mm, and to ∼85 V for the thickest film (1.15 mm). This monotonic decrease indicates that thinner dielectric layers enhance the effective electric field and interfacial potential difference generated during contact electrification. The thickness effect is even more pronounced in the static-contact mode, where *V*oc decreases from approximately 160 (0.3 mm film) to about 10 V for the 1.15 mm film, demonstrating a much stronger sensitivity to dielectric thickness under pre-pressure conditions.

**Figure 9:**
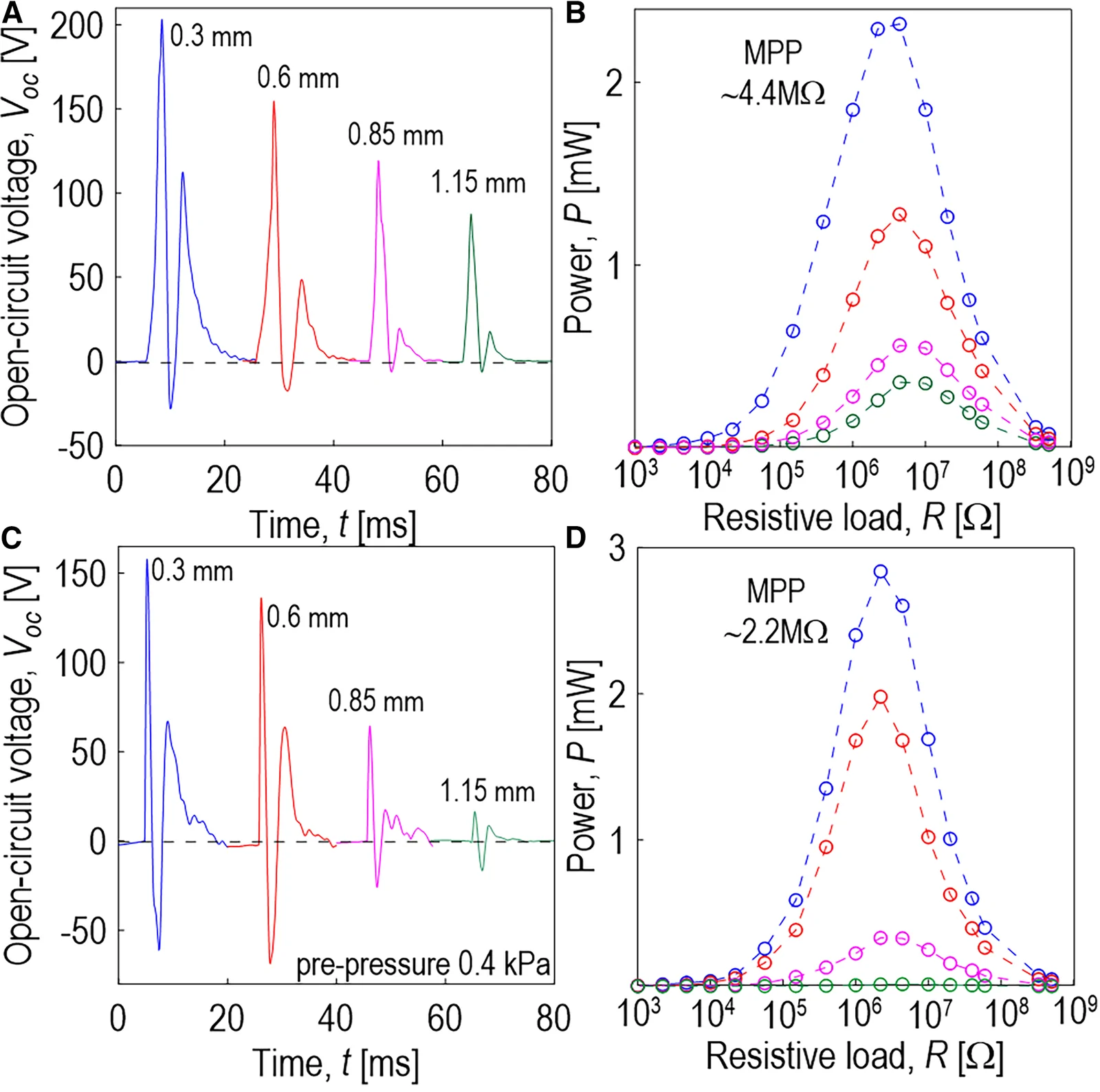
Increasing PVAc film thickness reduces voltage and power output of PVAc/Cu nanogenerator (A) Open-circuit voltage and (B) output power as functions of PVAc film thickness in contact–separation mode, (C) open-circuit voltage and (D) output power as functions of PVAc film thickness in static-contact mode at a pre-pressure of 0.4 kPa (1 Hz, 6.5 kPa, 18 cm^2^).

The load-dependent power output exhibits characteristic maxima at external resistances of approximately 4.4 and 2.2 MΩ in the contact-separation and static-contact modes, respectively, corresponding to the internal impedances of the devices (Figure 9). In the contact-separation regime, the peak power decreases progressively from 2.31 (0.3 mm) to 1.28 (0.6 mm), 0.56 (0.85 mm), and 0.36 mW (1.15 mm). In contrast, under static-contact operation at a pre-pressure of 0.4 kPa, the thickness dependence is markedly stronger: the peak power decreases from 2.84 (0.3 mm) to 1.98 mW (0.6 mm), followed by a sharp drop to 0.33 mW (0.85 mm) and to nearly negligible output (0.015 mW) for the 1.15 mm film.

The substantially stronger thickness sensitivity observed in the static-contact regime suggests that deformation-related interfacial mechanisms play a significant role under pre-pressure operation. Although strain gradients were not directly measured, the dramatic power suppression with increasing thickness is consistent with a deformation-mediated contribution to the mechano-electric response.

After measurements performed in the contact/separation mode, a modified configuration with a fixed air gap was used. The measurements were performed by adjusting the air gap using calibrated spacers after 60 triboelectric contact-separation cycles. The output voltage of 24 V, induced when the tribopair contacted and separated, dropped to 10.2 V at an air gap of 50 μm, 7.9 V at 100 μm, and 3.5 V at 550 μm to 0.7 V at a distance of 1.3 mm (Figure 10).

**Figure 10:**
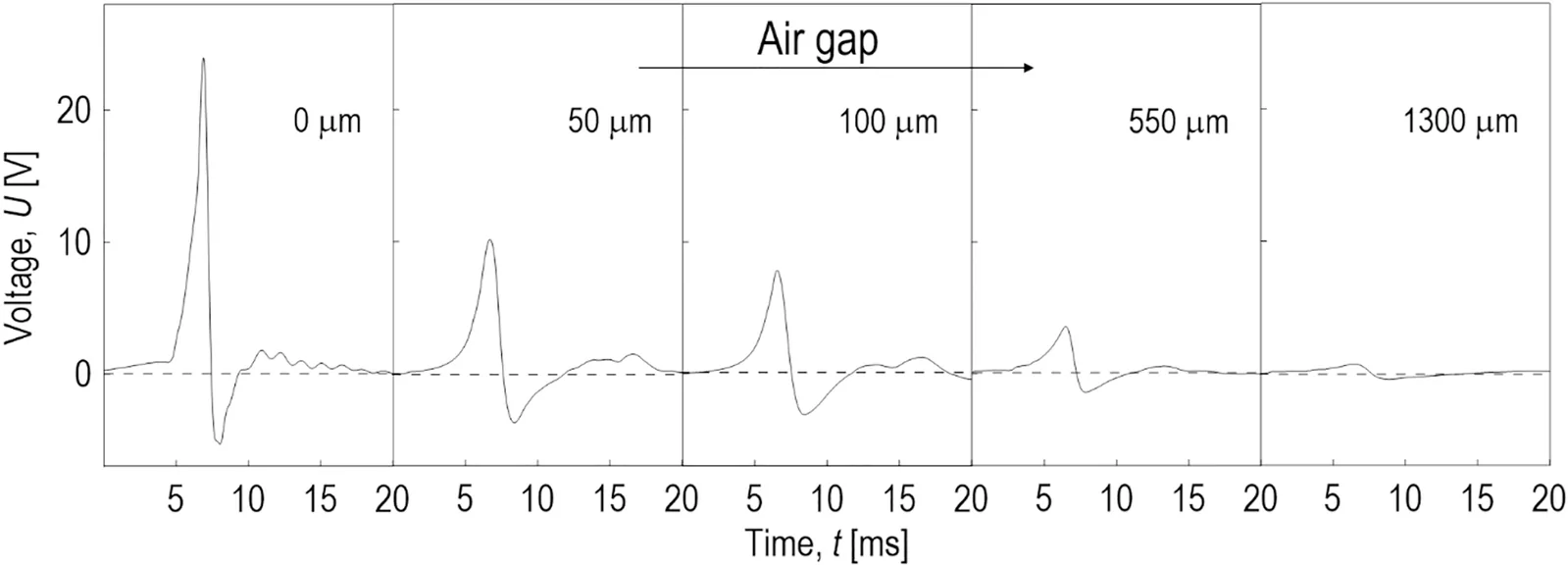
Air-gap-dependent output voltages of PVAc/Cu triboelectric pair (1 MΩ, 1 Hz, 6.5 kPa, 18 cm^2^)

According to cyclic experiments (Figure 11), a gradual increase in output voltage was observed during the initial contact cycles, eventually stabilizing around 25 V. This saturation behavior suggests a charge accumulation process. Although PVAc is not a typical electret material, its ability to retain charge-related states over time resembles electret-like behavior. The initial voltage response observed before mechanical contact results from an electrostatic induction between the polarized PVAc surface and Cu electrode. These contributions are consistent with recent discussions on pre-contact polarization phenomena in dielectric materials.^25^ Jeong et al.^25^ recently examined the origin of “non-contact” electric responses in TENG systems, proposing that such signals may result from electrostatic induction or residual polarization prior to a physical contact. Their study combines simulations and experiments to show that such pre-contact signals originate from electrostatic induction caused by charge redistribution, changes in capacitance, or residual surface polarization. This interpretation supports the idea that dielectric materials can exhibit a measurable electrical response purely due to electrostatic interactions, without requiring charge transfer through direct contact.

**Figure 11:**
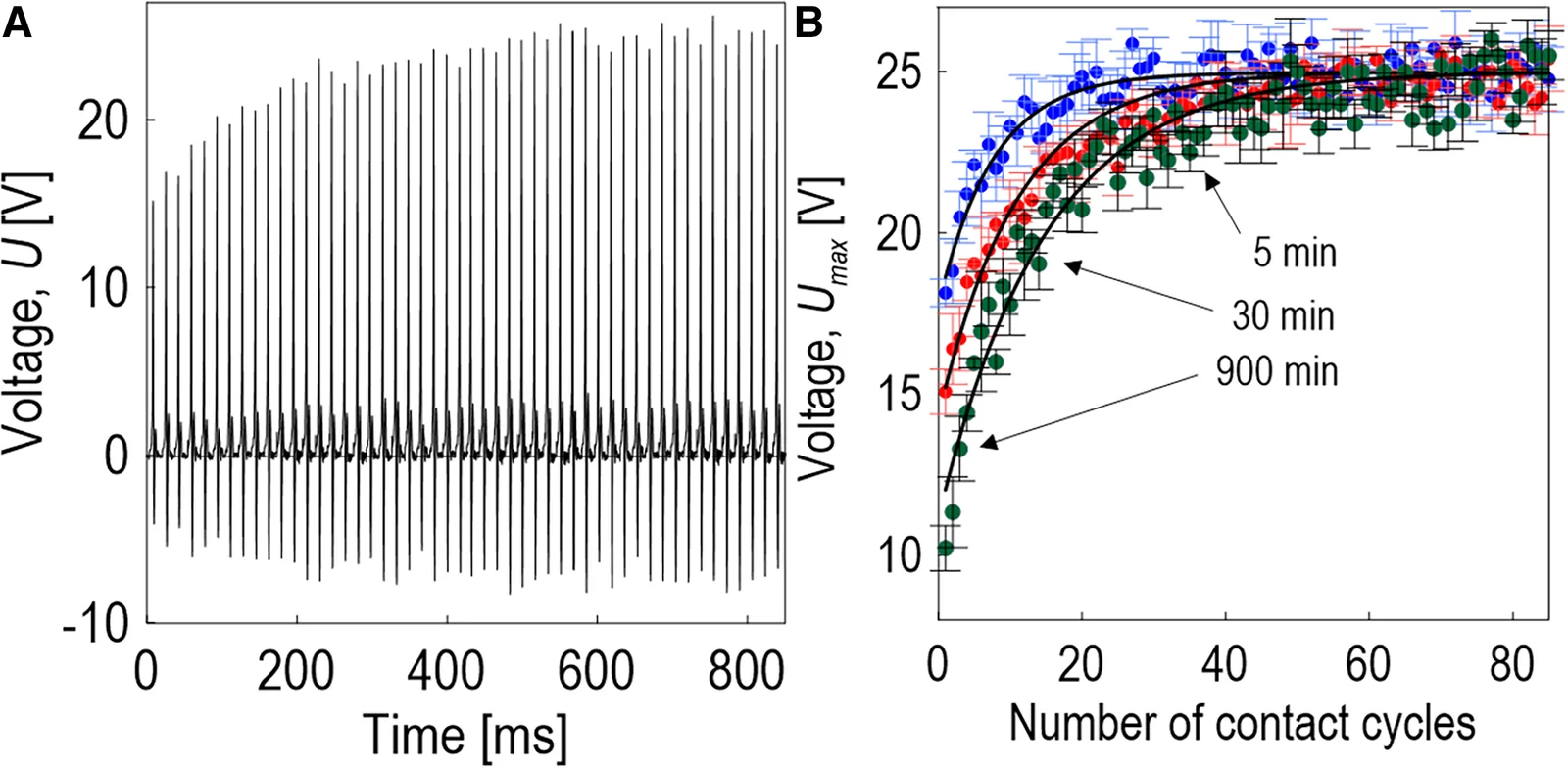
Charge accumulation and time-dependent relaxation of PVAc/Cu (A) Output voltage and (B) evolution of maximum output voltage of PVAc/Cu tribopair during contact-separation cycling at delay times of 5 (blue), 30 (red), and 900 (green) min (1 MΩ, 1 Hz, 6.5 kPa, 18 cm^2^). Error bars are reported as the mean ± standard deviation of seven repeated measurements (*n* = 7).

Further experimental evidence comes from the work of Šutka et al.,^26^ who systematically compared the behavior of poled and unpoled PVDF films. In their compressive triboelectric experiments, the unpoled samples exhibited no measurable response in non-contact mode, while poled PVDF produced clear voltage and current signals. This strongly supports the hypothesis that pre-polarization (e.g., via poling or mechanical activation) enables surface potential or internal dipolar alignment that contributes to the observed output even in the absence of physical contact. A similar conclusion was reported in a hybrid tribo-piezoelectric system, where poled ferroelectric layers significantly enhanced the output signal compared to unpoled materials.^27^

As also shown in Figure 11, the output voltages gradually increased during the contact cycles after predefined delay intervals following the first saturation phase. These delay times (5, 30, and 900 min) represent periods during which the device was left without mechanical stimulation. In each case, the output voltage was initially reduced after the delay but subsequently increased again over the course of several cycles. The longer the delay time was, the more pronounced the voltage decay was. This behavior confirms that the observed retention originates from slowly decaying trapped charges and dipolar alignment within the dielectric, resembling electret-like relaxation rather than full recharging.

The output voltage (*U*) exhibits a steep decrease with increasing gap (*d*), which is well described by a general inverse power-law dependence in the form *U* ∝ 1/*d*^1.6^, consistent with electrostatic induction influenced by the dielectric layer (Figure 12). Extrapolation of the fit toward zero separation yields a voltage of approximately 26 V, closely matching the value obtained in the contact-separation mode.

**Figure 12:**
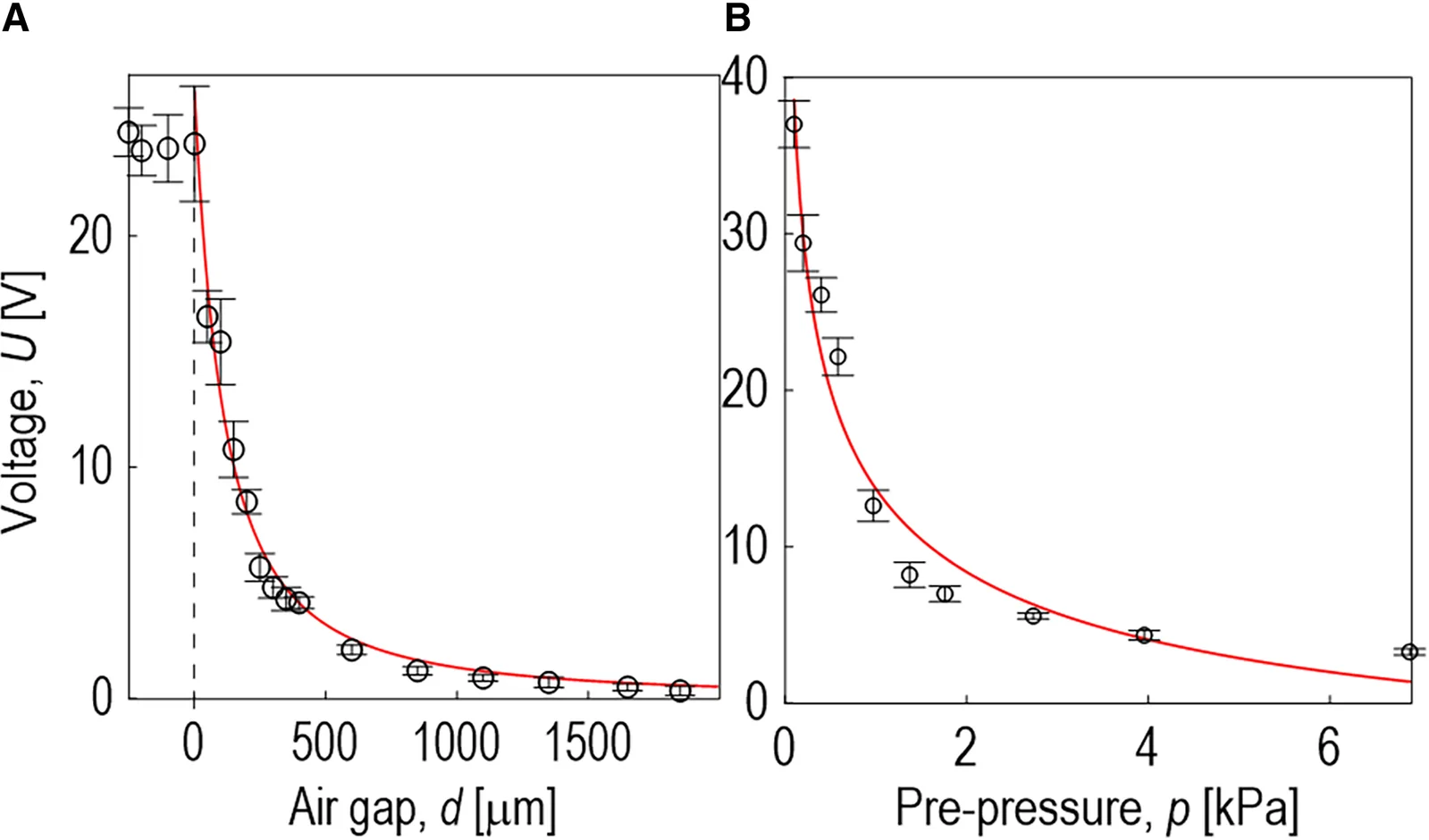
Output voltage of PVAc/Cu nanogenerator decreases with increasing air gap and static pre-pressure (A) Output voltage as a function of air gap in contact–separation/air-gap mode. (B) Output voltage as a function of pre-pressure in static-contact mode (1 MΩ, 1 Hz, 6.5 kPa, 18 cm^2^).

The dependence of the output voltage on the applied pre-pressure exhibited a monotonic logarithmic-type decrease, consistent with the qualitative prediction of the Greenwood-Williamson (GW) contact model.^28^ According to the GW description, the mean interfacial separation between rough surfaces decreases approximately logarithmically with the applied load, leading to a progressive reduction of the effective deformation amplitude during impact. The residual voltage response at increased pre-pressures indicated that a finite interfacial gap persists, with the actual contact area remaining limited to only a few percent of the nominal surface area—as predicted by the GW model. This partial-contact regime still allows minor local compression of asperities during the impact pulse, giving rise to a measurable voltage response. A similar logarithmic dependence was previously observed for biaxially oriented polypropylene films under static-contact loading,^29^ confirming that the GW-type interfacial behavior governs the pressure dependence of polymer-metal contact systems. Consistent with the literature,^17^ the surface roughness of the Cu electrode may generate localized deformation and induce polarization in the PVAc film molded onto the opposing electrode.

The output voltage generated after impact loading by a linear solenoid was found to depend on the initial pre-pressure between the Cu and PVAc electrodes. Prior to impact, the electrodes were brought into contact at different pre-pressures to assess the effect of PVAc surface deformation caused by the rigid and rough Cu surface. The voltage response during subsequent compression was more pronounced at lower pre-pressures, which preserved surface irregularities and allowed for more local deformations. This may have enhanced the mechano-electric response through a combination of different electrification mechanisms; e.g., a pre-pressure of 0.1 kPa resulted in a voltage of 37 V, while at 7 kPa, the output dropped to 2.4 V.

Triboelectric and static-contact (pre-pressure) modes were also used to evaluate the electrification between identical polymer surfaces (PVAc/PVAc) to assess whether a measurable signal persists even without a chemically dissimilar tribopair and/or surface topography. Same-material electrification has been reported in the literature^5^^,^^18^ and can be relevant for practical stacked/laminated polymer devices. When both electrodes were coated with PVAc, a measurable voltage was still observed upon mechanical excitation, as shown in Figure 13, suggesting that same-material electrification and induction/polarization effects can contribute under our conditions and contributions from deformation-driven polarization cannot be excluded.

**Figure 13:**
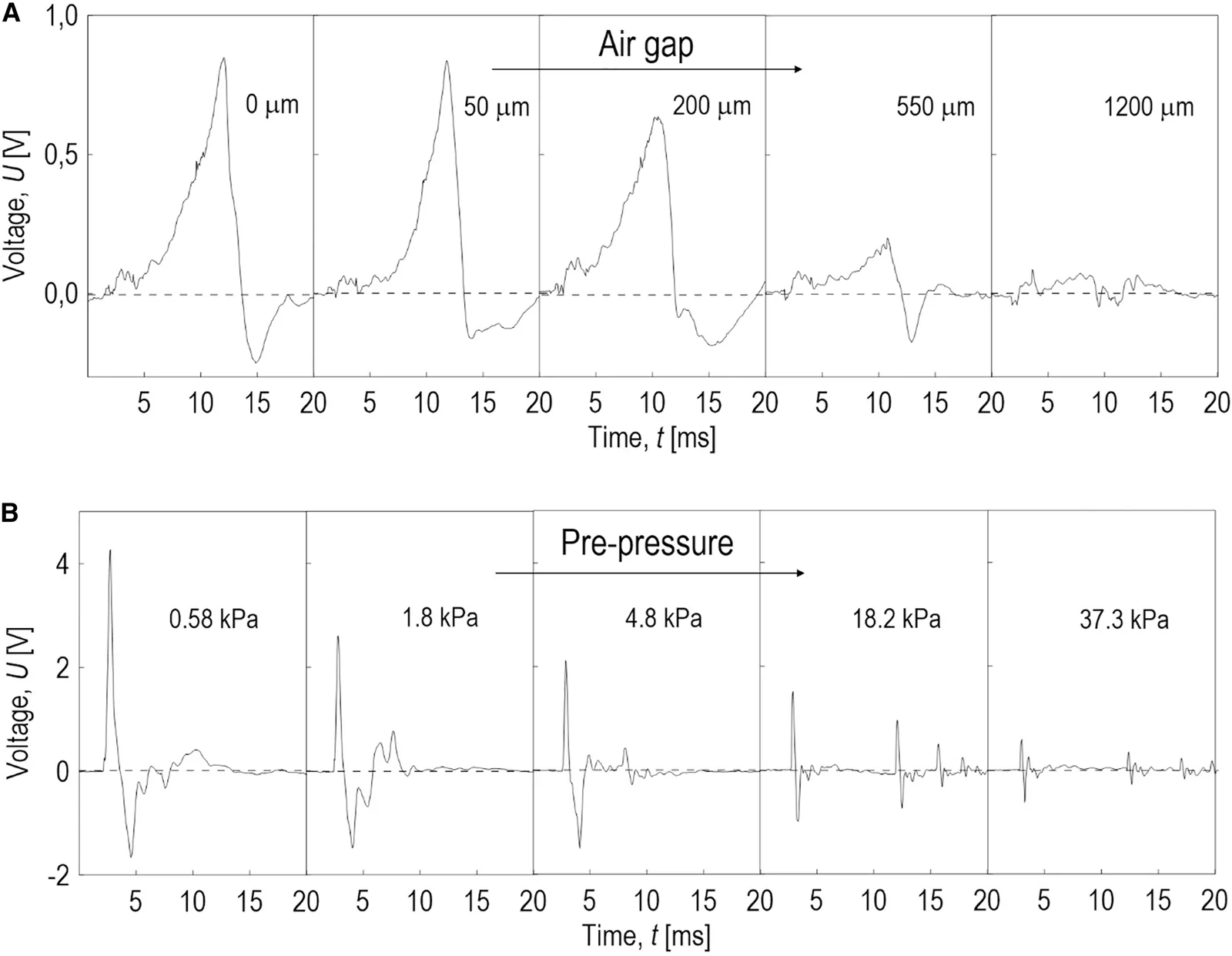
Identical PVAc/PVAc interfaces generate a measurable voltage response in static-contact operation Output voltages generated by linear solenoid in static-contact mode at various (A) air gaps and (B) pre-pressures (1 MΩ, 1 Hz, 6.5 kPa, 18 cm^2^).

## Discussion

In this study, we established PVAc as an understudied, tribopositive soft adhesive for mechano-electric energy conversion operating in contact-separation, air-gap, and static-contact (pre-pressure) configurations. The combined analysis of load dependence, air-gap scaling, pre-pressure response, and film thickness variation demonstrates that triboelectric charge transfer, electrostatic induction from residual polarization, and deformation-related interfacial mechanisms act cooperatively rather than independently. Although a distinct flexoelectric contribution cannot yet be quantitatively isolated, the strong thickness sensitivity and pressure dependence observed in static-contact operation are consistent with deformation-mediated interfacial effects.

Contact-separation and static-contact modes revealed at 1 Hz open-circuit voltage, short-circuit current density, and power density above 160 V, 1.8 μAcm^−2^, and 0.07 mWcm^−2^ in contact-separation and 110 V, 2.6 μAcm^−2^, and 0.07 mWcm^−2^ at low pre-pressures, respectively, together with a stable operation over 10^4^ cycles. The shift of the maximum power point from 4.4 (contact-separation) to 2.2 MΩ (static-contact) indicates different effective internal impedances and supports the distinct mechano-electric dynamics in the two regimes. Furthermore, static pre-pressure can either enhance or suppress the output depending on its magnitude, highlighting the critical role of pressure-controlled interfacial mechanics.

Benchmarking against state-of-the-art tribopositive polymers (Table 1) shows that PVAc provides competitive performance without poling, nanostructuring, or conductive fillers, while benefiting from strong adhesion and straightforward melt processing.

**Table 1:** Benchmarking of PVAc to tribopositive polymers comparing open-circuit voltage, short circuit current, short-circuit current density, power density, stability, frequency, operating force, device area, and electrode material

Tribopositive polymer	Pair	*V*oc (V)	*I*sc (μA)	*J*sc (μA/cm^2^)	*PD* (mW/cm^2)^	Stability (no. of cycles)	*f* (Hz)	*F* (N)	*A* (cm^2^)	Elect.
**PVAc**	**PVAc/Cu**	**302**	**38.5**	**2.6**	**0.21**	**10,000**	**10**	**12**	**18**	**Cu**
PVAc^30^	PVAc/STG	750	235	10	1.5	10,000	1	10	24	steel
PVA^31^	PVA/PP	22	1.72	–	0.002	–	6	10	25	Al
PVA^32^	PVA/SR	80	–	3	0.4	–	1	50	4	Al
PVA + CF^32^	PVA + CF/SR	125	–	8	1.28	–	1	50	4	Al
PVA^33^	PVA/PTFE	128	2.83	–	0.024	–	3	5	16	Cu
PVA^34^	PVA/silicone	641	189	11.8	1.08	–	2	10	16	Al
PEO + Ag/SiO_2_^35^	Ag/SiO_2_-PEO/SR	95	10	–	0.15	10,000	3	–	4	–
PEO^36^	PEO/PDMS	485	–	8.5	4	–	5	50	4	Al
PA6^36^	PA6/PDMS	315	–	3	1.8	–	5	50	4	Al
PA6,6^37^∗	PA6,6/PTFE	1,300	14.6	0.41	0.042	–	–	–	35.5	–
PA11^38^	PA11/BaTiO_3_-PVDF	280	12	0.75	0.145	–	–	–	16	Al
PA11 + EC^39^	PA11 + EC/PTFE-PVDF	212	18.5	18.5	0.176	100,000	3	50	6.25	ITO

Mode: contact-separation, except ∗lateral sliding. *V*oc, open-circuit voltage; *I*sc, short circuit current; *J*sc, short-circuit current density; *PD*, power density; *f*, frequency; *F*, operating force; *A*, device area.

Bold entries highlight the results of this study.

### Limitations of the study

This study relies exclusively on macroscopic electrical measurements and does not include direct, spatially resolved probing of strain gradients, surface potential, or local charge distribution at the PVAc/Cu interface. Therefore, a distinct flexoelectric contribution cannot be quantitatively isolated from triboelectric charge transfer and electrostatic induction, and it is discussed only as a plausible mechanism consistent with the literature. The study does not include a wearable prototype tested in long-term environmental (temperature and humidity) testing; thus, the practical durability and performance of PVAc-based devices under real operating conditions remain to be established in future work.

## Resource availability

### Lead contact

Requests for further information, resources, and reagents should be directed to and will be fulfilled by the lead contact, Berenika Hausnerova (hausnerova@utb.cz).

### Materials availability

This study did not generate new unique reagents.

### Data and code availability

• All data reported in this paper will be shared by the lead contact upon request.
• This paper does not report original code.
• Any additional information required to reanalyze the data reported in this paper is available from the lead contact upon request.

## Acknowledgments

This work was supported by the Ministry of Education, Youth, and Sports of the Czech Republic within the framework of DKRVO (RP/CPS/2024-2028/005).

## Author contributions

P.S., conceptualization, methodology, validation, formal analysis, investigation, data curation, visualization, and writing – original draft; P.R., conceptualization and writing – review and editing; B.H., conceptualization, resources, visualization, writing – review and editing, and funding. All authors have read and agreed to the content of the manuscript.

## Declaration of interests

The authors declare no competing interests.

## STAR★Methods

### Key resources table

**Table:** 

REAGENT or RESOURCE	SOURCE	IDENTIFIER
**Chemicals, peptides, and recombinant proteins**		

Poly(vinyl acetate) (PVAc), Mw 500 kg mol^−1^	Sigma-Aldrich	387932-500G
Copper foil on glass-epoxy substrate	N/A	Cu foil thickness: 35 μm; glass-epoxy substrate thickness: 200 μm

**Deposited data**		

Raw data reported in this study	This paper	Available from the lead contact upon request

**Software and algorithms**		

Grapher Software	Golden Software, LLC	Version 10.0

**Other**		

Differential scanning calorimeter DSC 1	PerkinElmer, Waltham, MA, USA	DSC 1
Dynamic mechanical analyzer	Mettler Toledo, Switzerland	DMA/SDTA861e
Broadband dielectric/impedance analyzer	Novocontrol Technologies GmbH & Co. KG, Montabaur, Germany	DEA
3D optical scanner/profilometer with Coherence Scanning Interferometry technology	AMETEK, Inc., Middlefield, CT, USA	Zygo NewView 8000
Arduino controller	Arduino	N/A
Analog converter for strain-gauge measurement	VTS Zlín s.r.o., Zlín, Czech Republic	TZA11410
Oscilloscope	Keysight, Santa Rosa, CA, USA	Infiniivision 1000 X-Series; DSOX1204A; 4-channel, 100 MHz
Charge sensor	Vernier Edufor s.r.o., Prague, Czech Republic	GRD-BTA charge sensor
LabQuest interface system	Vernier Edufor s.r.o., Prague, Czech Republic	LabQuest
3D-printed flexible nanogenerator assembly element, elastic acrylic rubber	This paper	Custom-made
Duralumin pressure-distribution plate	This paper	Custom-made
PVAc/Cu triboelectric nanogenerator	This paper	Custom-made

### Experimental model and study participant details

This session has been omitted as this physical-science study does not involve biological models.

### Method details

#### Materials

PVAc was purchased from Sigma-Aldrich (M_w_ = 500 kg/mol). Cu foil on a glass epoxy substrate was used for the electrodes (the thicknesses of the Cu foil and the substrate were 35 μm and 200 μm, respectively). PVAc was pressed onto the electrode in the molten state (140°C) to a 0.6 mm thick film adhering precisely to the Cu surface of the electrode. Additional films with thicknesses of 0.3, 0.85, and 1.15 mm were prepared for the thickness-dependence measurements.

#### Characterization methods

Differential scanning calorimetry (DSC 1, PerkinElmer, Waltham, MA, USA) was used to evaluate the glass transition temperature, found to be approximately 35°C, from the maximum in the heat flow curve. The thermomechanical properties of PVAc films were determined using a dynamic mechanical analyzer (DMA/SDTA861e, Mettler Toledo, Switzerland) in tensile mode at a fixed frequency of 1 Hz, in a temperature range from 0°C to 60°C, with a heating rate of 1 °C/min.

The dielectric properties of the PVAc films were measured using a broadband dielectric/impedance analyzer (DEA, Novocontrol Technologies GmbH & Co. KG, Montabaur, Germany) in a frequency range from 1 Hz to 10 MHz at room temperature (22°C). A circular sample with a defined thickness of 0.6 mm was placed between two gold-plated electrodes (20 mm in diameter).

A 3D scanner (Zygo NewView 8000, AMETEK, Inc., Middlefield, Connecticut, USA) with Coherence Scanning Interferometry (CSI) technology, which combines VSI - Vertical Scanning Interferometry and PSI - Phase Shifting Interferometry was used to determine the standard surface roughness parameters Sa, arithmetic mean height; Sq, root-mean-square height; and Sz, maximum height according to the ISO 21920-2 standard (2023).

#### Experimental set-up

Using stereolithography, a flexible nanogenerator assembly element was printed from elastic acrylic rubber (Figure S1). The plain Cu electrode was glued to the top plate of the 3D-printed rubber element, whereas the Cu electrode with the PVAc film was attached to its bottom plate. The lateral elastic connection acted as a spring, ensuring mutual movement of the plates. The upper duralumin plate (a high-strength aluminum alloy primarily composed of aluminum, copper, manganese, and magnesium) ensured an even distribution of pressure exerted by a DC 12 V linear solenoid and also served as mechanical protection against wear of the printed nanogenerator assembly. The PVAc film had dimensions of 60 × 30 mm, corresponding to an active area of 18 cm^2^. The reversible linear solenoid was controlled using an Arduino hardware program. The strain gauge measuring the stress during PVAc solenoid loading was powered using an analog converter (TZA11410, VTS Zlin s.r.o., Zlin, Czech Republic) with an output of ±20 mA and a 24V DC power supply. The maximum stress induced during the solenoid compression pulse was 6.5 kPa (corresponding to ∼11.7 N) and the displacement speed was ∼0.26 m s^−1^. During each pulse, the solenoid maintained the applied compression at the closest plate position for approximately 0.5 s at 1 Hz and 50 ms at 10 Hz, followed by a rapid return to the initial position. All measurements were performed under air-conditioned laboratory conditions at approximately 22.5°C and 38% relative humidity.

An oscilloscope (Infinivision 1000 x-series, 4ch, 100 MHz, DSOX1204A, Keysight, Santa Rosa, CA, USA) equipped with a high-impedance differential voltage probe was used to measure the open-circuit voltage (*V*oc) across the device electrodes. For load-dependent measurements, external resistive loads, *R,* ranging from 1 kΩ to 500 MΩ were connected across the electrodes. For each external load and operating condition, the voltage waveform was recorded and the corresponding peak voltage amplitude, *V*, was determined. Representative voltage–time profiles are shown in the figures to illustrate the characteristic electrical response. Finally, the charge generated per cycle was measured using an electroscope (GRD-BTA charge sensor) connected to a LabQuest interface system (Vernier Edufor s.r.o.; Prague, Czech Republic).

In the contact–separation mode, the electrodes were initially separated by approximately 6 mm. Each solenoid pulse brought the Cu electrode into direct contact with the PVAc surface and maintained the contact under the applied pressure for approximately 0.5 s at 1 Hz and 50 ms at 10 Hz, followed by the rapid macroscopic separation after the pulse.

In the air-gap induction mode, two calibrated steel spacers prevented direct contact between the electrodes and defined their minimum separation during each solenoid pulse. In the unloaded position, the electrode gap was approximately 6 mm. Air-gap measurements were performed after triboelectric preconditioning of the PVAc/Cu pair consisting of 60 contact–separation cycles at an external resistance of 1 MΩ, a frequency of 1 Hz, an applied pressure of 6.5 kPa, and an active area of 18 cm^2^.

In the static-contact (pre-pressure) mode, a defined static pre-pressure was applied using calibrated weights placed on the upper duralumin plate and maintained throughout the measurement. This configuration ensured continuous contact between the electrodes and suppressed macroscopic approach–separation, thereby emphasizing deformation-driven interfacial processes. Each solenoid pulse superimposed an additional dynamic compression on the preloaded PVAc film. Measurements were performed at several pre-pressure levels and external resistive loads.

### Quantification and statistical analysis

For each external load and operating condition, seven repeated measurements were performed (*n* = 7, where *n* represents independent repeated electrical measurements under the same experimental conditions). Where error bars are shown, data are presented as mean ± standard deviation. The current, *I*, was subsequently calculated according to Ohm’s law as *I = V/*R, where *V* represents peak voltage amplitude and *R* is the resistance*.* Representative voltage–time profiles are shown in the figures to illustrate the characteristic electrical response. Where error bars are provided, the quantitative data are reported as the mean ± standard deviation of seven repeated measurements (*n* = 7). The current density (*Jsc*) was determined by normalizing the calculated current to the effective active area of the device (18 cm^2^). The instantaneous power (*P*) was calculated as *P* = *V*^2^/*R*, and the power density (*PD*) was obtained by dividing the power by the active device area. The triboelectric charge density (TECD) was calculated by normalizing the measured charge to the active area, and the values are reported as mean values from seven measurements (*n* = 7). Data processing and plotting were performed using Grapher Software, version 10.0 (Golden Software, LLC).
