# Supplementary material for: Mechano-electric response of tribopositive, fluoropolymer-free polyvinyl acetate nanogenerators in multiple contact electrification
Source: iScience. 2026 Oct 12;29(11):117808. doi: 10.1016/j.isci.2026.117808 (PMC13682346; doi:10.1016/j.isci.2026.117808)

iScience, Volume *29*

Supplemental information

Mechano-electric response of tribopositive, fluoropolymer-free polyvinyl acetate nanogenerators in multiple contact electrification

Petr Slobodian, Pavel Riha, and Berenika Hausnerova

Figure S1. Design and dimensions of the 3D-printed nanogenerator assembly. (A) Schematic representation of the assembly used for the measurements in different operating modes. (B) Construction details and dimensions, in mm, of the 3D-printed flexible nanogenerator body.

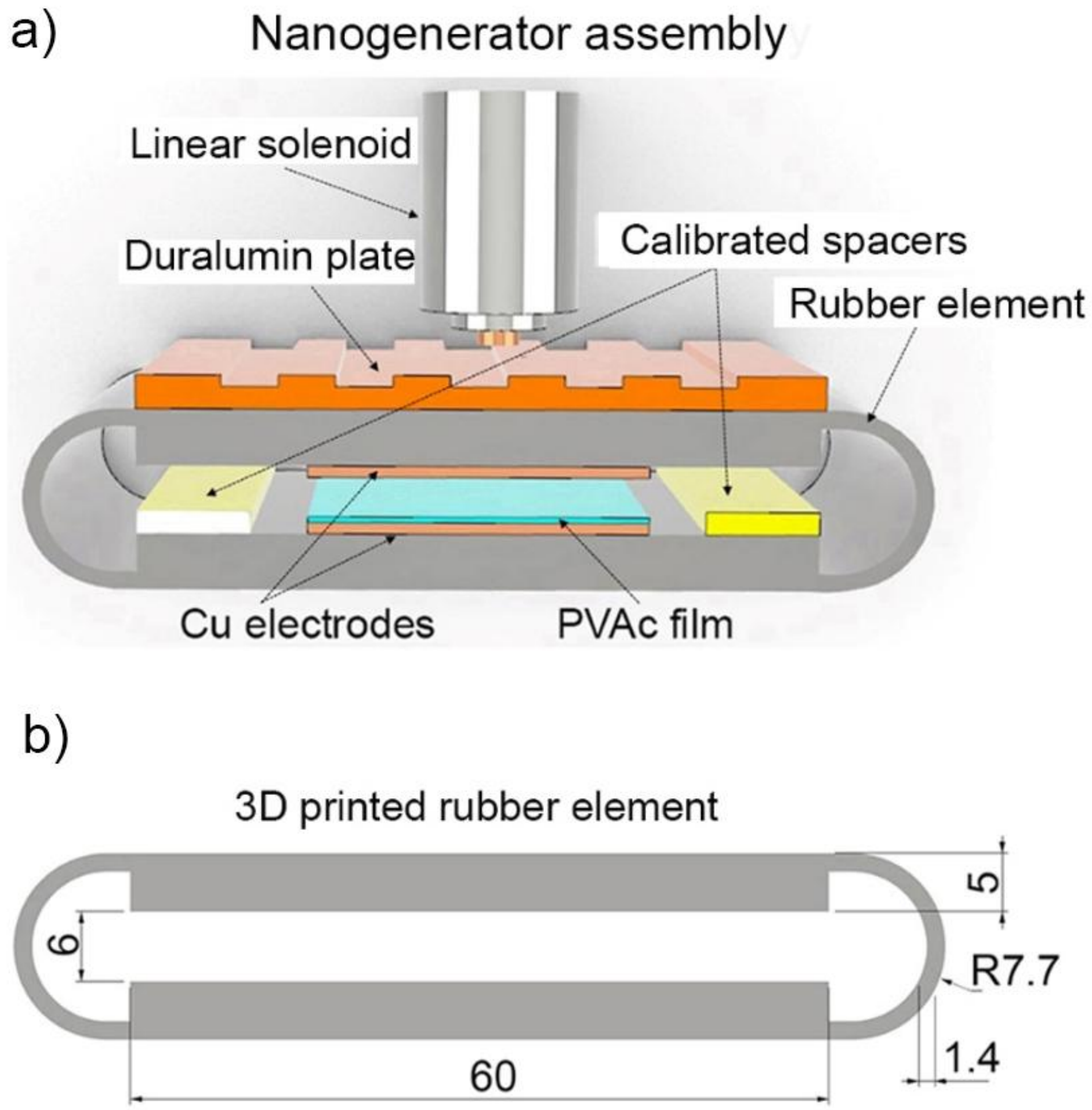

Supplement: Supplementary file 1 [file mmc1.pdf]
